# Macroevolution of thermal tolerance in intertidal crabs from Neotropical provinces: A phylogenetic comparative evaluation of critical limits

**DOI:** 10.1002/ece3.2741

**Published:** 2017-03-31

**Authors:** Samuel C. Faria, Rogério O. Faleiros, Fábio A. Brayner, Luiz C. Alves, Adalto Bianchini, Carolina Romero, Raquel C. Buranelli, Fernando L. Mantelatto, John C. McNamara

**Affiliations:** ^1^ Departamento de BiologiaFaculdade de FilosofiaCiências e Letras de Ribeirão PretoUniversidade de São PauloRibeirão PretoBrazil; ^2^ Centro de Pesquisas Aggeu MagalhãesFiocruzRecifeBrazil; ^3^ Laboratório de Imunopatologia Keizo AsamiLIKA/UFPERecifeBrazil; ^4^ Instituto de Ciências BiológicasUniversidade Federal do Rio GrandeRio GrandeBrazil; ^5^ Centro Austral de Investigaciones CientíficasCADIC‐CONICETUshuaiaArgentina; ^6^ Centro de Biologia MarinhaUniversidade de São PauloSão SebastiãoBrazil; ^7^ Present address: Instituto de Ciências BiológicasUniversidade Federal do Rio GrandeRio GrandeBrazil

**Keywords:** 16Smt gene, comparative methods, evolutionary physiology, temperature, thermal limits, zoogeographical province, Crustacea, Decapoda

## Abstract

Thermal tolerance underpins most biogeographical patterns in ectothermic animals. Macroevolutionary patterns of thermal limits have been historically evaluated, but a role for the phylogenetic component in physiological variation has been neglected. Three marine zoogeographical provinces are recognized throughout the Neotropical region based on mean seawater temperature (*T*_m_): the Brazilian (*T*_m_ = 26 °C), Argentinian (*T*_m_ = 15 °C), and Magellanic (*T*_m_ = 9 °C) provinces. Microhabitat temperature (MHT) was measured, and the upper (UL_50_) and lower (LL_50_) critical thermal limits were established for 12 eubrachyuran crab species from intertidal zones within these three provinces. A molecular phylogenetic analysis was performed by maximum likelihood using the 16S mitochondrial gene, also considering other representative species to enable comparative evaluations. We tested for: (1) phylogenetic pattern of MHT, UL_50_, and LL_50_; (2) effect of zoogeographical province on the evolution of both limits; and (3) evolutionary correlation between MHT and thermal limits. MHT and UL_50_ showed strong phylogenetic signal at the species level while LL_50_ was unrelated to phylogeny, suggesting a more plastic evolution. Province seems to have affected the evolution of thermal tolerance, and only UL_50_ was dependent on MHT. UL_50_ was similar between the two northern provinces compared to the southernmost while LL_50_ differed markedly among provinces. Apparently, critical limits are subject to different environmental pressures and thus manifest unique evolutionary histories. An asymmetrical macroevolutionary scenario for eubrachyuran thermal tolerance seems likely, as the critical thermal limits are differentially inherited and environmentally driven.

## 1 Introduction

Thermal tolerance in animals depends on systemic through subcellular functions of resistance, entailing complex physiological interactions that underpin most biogeographical distribution patterns of life on Earth. Such tolerance results from physiological mechanisms usually manifested under extreme environmental conditions, which can be considered as descriptors of spatial discontinuities among ectotherms. Critical temperatures are those at which the loss of essential motor capability occurs, resulting in disorganized locomotor activity, affecting an organism's ecological function (Cowles & Bogert, 1944; Lutterschmidt & Hutchison, 1997; Sunday, Bates, & Dulvy, 2010). Survival under such extreme conditions is constrained by the availability of energy generated anaerobically (Zielinski and Pörtner, 1996; Sommer, Klein, & Pörtner, 1997), in addition to the mobilization of heat‐shock proteins and antioxidant defenses (Heise, Puntarulo, Nikinmaa, Abele, & Pörtner, 2006; Tomanek, 2005).

An association between thermal tolerance and latitude has been explored historically, as has the adjustability of critical or lethal thermal limits on acclimation and/or acclimatization (Addo‐Bediako, Chown, & Gaston, 2000; Compton, Rijkenberg, Drent, & Piersma, 2007; Cruz, Fitzgerald, Espinoza, & Schulte, 2005). This association is no exception among the Pancrustacea. In such arthropods, variability in thermal limits reflects the diversity of the microhabitat temperatures occupied: (1) along latitudinal gradients [e.g., fiddler crabs *Uca* (Vernberg & Tashian, 1959; Vernberg & Vernberg, 1967), diver beetles *Deronectes* (Calosi, Bilton, & Spicer, 2008; Calosi, Bilton, Spicer, Votier, & Atfield, 2010); (2) among different climatic niches [e. g. *Drosophila* flies (Kellermann, Loeschcke et al., 2012; Kellermann, Overgaard et al., 2012)]; (3) in vertical distribution within the intertidal zone [e.g., anomurans *Petrolisthes* (Stillman, 2002; Stillman & Somero, 2000) and crabs *Carcinus* and *Cancer* (Cuculescu, Hyde, & Bowler, 1995)]; and (4) within marine and freshwater environments [e. g. penaeoidean and caridean shrimps (Manush, Pal, Chatterjee, Das, & Mukherjee, 2004; Ravaux, Léger, & Rabet, 2012; Selvakumar & Geraldine, 2005)].

Upper critical thermal limits (UL_50_) can thus predict latitude, vertical position in an intertidal environment, or the biotope occupied by different species, as they correlate positively with microhabitat temperature and are associated with mechanisms of thermal tolerance. In contrast, lower critical thermal limits (LL_50_) are poorly explored in a latitudinal context, although there is a link between lower thermal resistance and higher latitudes (Demeusey, 1957; Tashian, 1956; Vernberg & Tashian, 1959; Vernberg & Vernberg, 1967). Lower limits seem to correlate better with ecological factors such as predation, competition, and substratum preference than with physiological limits (Jensen & Armstrong, 1991; Paine, 1974).

Most studies have characterized thermal tolerance in a species‐specific manner, or have compared tolerance over a broad range of taxonomic levels, neglecting the influence of the phylogenetic component on physiological variation. Interspecific comparisons must consider the phylogenetic relationships among species due to their lack of statistical independence owing to shared ancestry (Felsenstein, 1985; Garland, Bennett, & Rezende, 2005; Garland & Ives, 2000). Different lineages tend to evolve independently of one another, and physiological variation among species thus increases as a function of phylogenetic distance, rendering closely related species more similar for reasons of ancestry and not necessarily as a consequence of environmental pressures (Harvey & Pagel, 1991; Rezende & Diniz‐Filho, 2012). While it is essential to retrieve the evolution of thermal tolerance, the use of phylogenetic information when evaluating critical thermal limits across a large geographical distribution is rarely encountered in crustacean studies (see Stillman, 2002; Stillman & Somero, 2000).

The American continent is classified into 16 marine zoogeographical provinces, which are defined as part of the neritic zone characterized by a narrow range of water temperatures, and containing a fairly constant decapod crustacean fauna (see Boschi, 2000a,b). Specifically, the eastern coast of South America is divided into three provinces: (1) the Brazilian zoogeographical province, delimited by the mouth of Orinoco River, Venezuela (9° N), and extending to Cabo Frio/RJ, Brazil (23° S) (Briggs, 1974); (2) the Argentinian province, between Cabo Frio and Rawson, Argentina (43° S) (Cooke, 1895); and (3) the Magellanic province, from Rawson to Ushuaia, Argentina (55° S) (Carcelles & Williamson, 1951). The Brazilian (22–30 °C) and Magellanic (4–15 °C) provinces are more stenothermic than the Argentinian province (8–23 °C), because the first is dominated by the South Equatorial Current (Thurman, Faria, & McNamara, 2013), and the second by the homogeneous mass of subantarctic waters (Boschi, 2000a). The Argentinian province is the most eurythermic region, characterized by a mixture of cold water from the Malvinas Current and warmer waters from the Brazil Current, and tends to include species more tolerant of temperature variation (Boschi, 2000a).

Here, we propose an evolutionary history of thermal tolerance in 12 intertidal, eubrachyuran crab species, selected based on their ample distribution across the three zoogeographical provinces along the eastern coast of South America. A phylogenetic analysis was performed using partial sequences of the 16S mitochondrial genes from the selected species, including other Brachyura and Anomura for comparative evaluation. We tested for: (1) phylogenetic patterns of microhabitat temperature (MHT), UL_50_, and LL_50_; (2) an effect of zoogeographical province on the evolution of both critical thermal limits; and (3) an evolutionary correlation between MHT and these limits. The macroevolutionary pattern of thermal tolerance is discussed in a biogeographical context, particularly regarding the phylogenetic and environmental components.

## 2 Material and Methods

### 2.1 Crab species and laboratory maintenance

The crab species chosen were collected from three distinct thermal provinces along the eastern coast of South America: (1) the Brazilian province (≈7.8° S/34.8° W, Ilha de Itamaracá or Itapissuma, PE, Brazil)—*Aratus pisonii* H. Milne Edwards, 1837; *Cardisoma guanhumi* Latreille, 1825; *Goniopsis cruentata* Latreille, 1803; *Ocypode quadrata* Fabricius, 1787; *Pachygrapsus transversus* Gibbes, 1850; *Uca maracoani* Latreille 1802–1803; and *Ucides cordatus* Linnaeus, 1763 [these species were held at the Universidade Federal de Pernambuco (Recife, PE, northeastern Brazil)]; (2) the Argentinian province (≈32.1° S/52.1° W, Rio Grande, RS, Brazil)—*Armases rubripes* Rathbun, 1897; *Neohelice granulata* Dana, 1851; and *Uca uruguayensis* Nobili, 1901 [these species were held at the Universidade Federal do Rio Grande (Rio Grande, RS, southern Brazil); and (3) the Magellanic province (≈53.2° S/67.2° W, Ushuaia, Tierra del Fuego, Argentina)—*Acanthocyclus albatrossis* Rathbun, 1898; and *Halicarcinus planatus* Fabricius, 1775 [these species were held at the Centro Austral de Investigaciones Científicas/Consejo Nacional de Investigaciones Científicas y Técnicas (CADIC/CONICET, Ushuaia, Argentina)].

**Figure 1:**
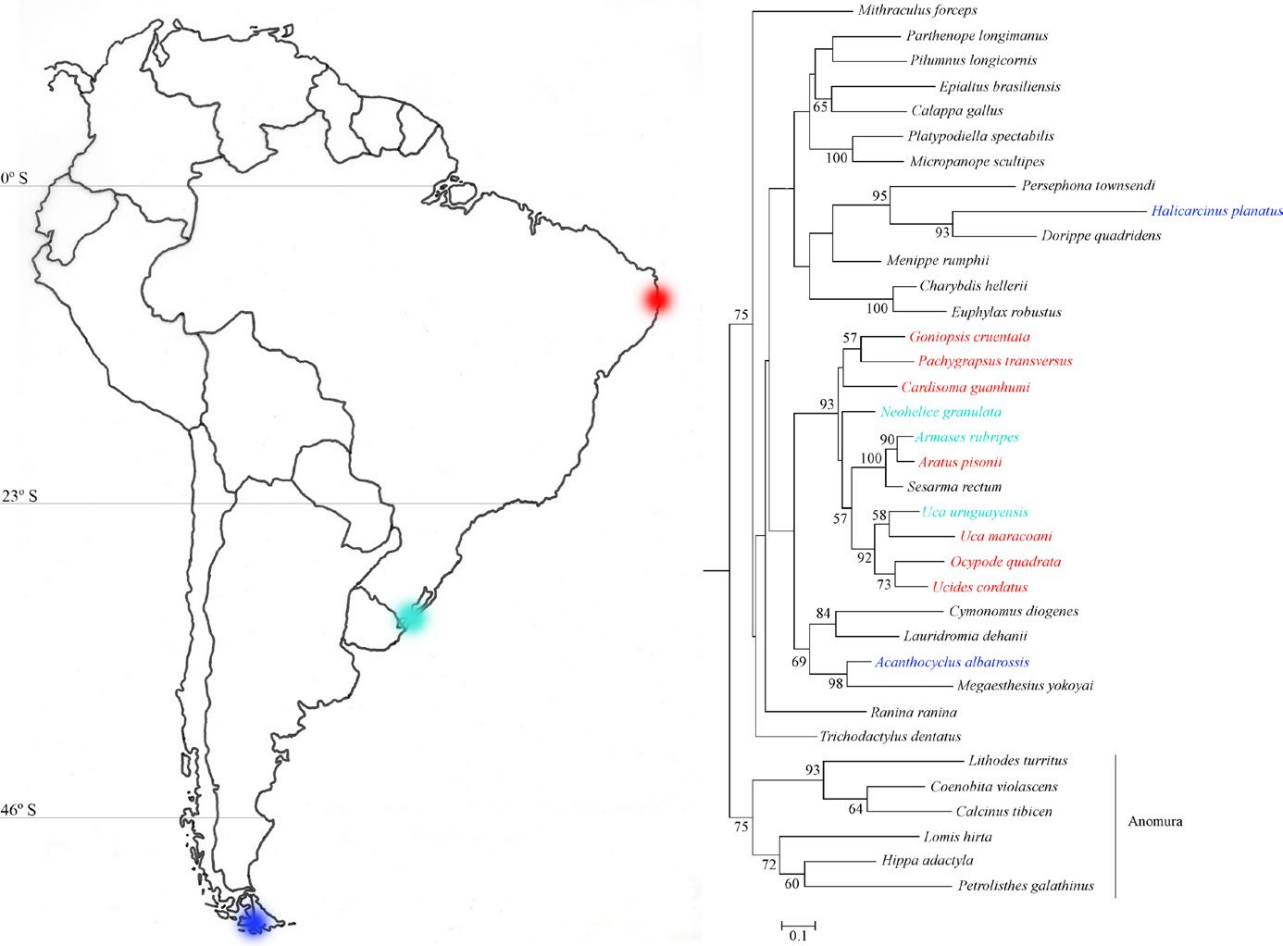
Collecting sites within the three zoogeographical provinces on the eastern coast of South American (sensu Boschi, 2000a) (*left*) and a phylogenetic hypothesis for selected brachyuran crab species (*right*). The Brazilian zoogeographical province is delimited by the mouth of Orinoco River, Venezuela (9° N), and Cabo Frio/RJ, Brazil (23° S); the Argentinian province lies between Cabo Frio and Rawson, Argentina (43° S); and the Magellanic province extends from Rawson to Ushuaia, Argentina (55° S). *Aratus pisonii*,* Cardisoma guanhumi*,* Goniopsis cruentata*,* Ocypode quadrata*,* Pachygrapsus transversus*,* Uca maracoani*, and *Ucides cordatus* were collected from the Brazilian province (red, ≈7.8° S/34.8° W, Ilha de Itamaracá or Itapissuma, PE, Brazil); *Armases rubripes*,* Neohelice granulata*, and *Uca uruguayensis* from the Argentinian province (cyan, ≈32.1° S/52.1° W, Rio Grande, RS, Brazil); and *Acanthocyclus albatrossis* and *Halicarcinus planatus* from the Magellanic province (blue, ≈53.2° S/67.2° W, Ushuaia, Tierra del Fuego, Argentina). The molecular phylogeny was generated using a maximum likelihood search method employing a partial sequence of the mitochondrial 16S gene (592 base pairs, GTR + Γ + I model) for 36 brachyuran and anomuran (outgroup) species. The final alignment of the fragments consisted of 15 novel sequences and 21 sequences obtained from NCBI GenBank. The nodal values represent clade support (bootstrap)

Approximately 80 adult, intermolt crabs of either sex from each of the 12 species were collected manually from mangroves, salt marshes, and sandy or rocky beaches along the eastern coast of South America (Figure 1). Substrate temperature was measured using an infrared, digital thermometer (Icel TD‐965, 0.1 °C precision) during crab collections (7 ≤ *N* ≤ 15 measurements per species). Specimens were obtained during the morning low tides at the end (February–March) of the summers of 2013 and 2014. In the laboratory, the crabs were held in plastic boxes containing a 4‐ to 8‐mm‐deep film of seawater in incubators or a water bath (Fanem 347 DCG; Solab SL 224; Polystat 12002, Palmer Instrument Company) at the annual mean seawater temperature of the respective provinces (sensu Boschi, 2000a,b, Boschi & Gavio, 2005) for 3 days prior to experiments, i. e. Brazilian province, 26 °C, 12‐hr light/12‐hr dark photoperiod; Argentinian province, 15 °C, 14‐hr light/10‐hr dark photoperiod; and Magellanic province, 9 °C, 14‐hr light/10‐hr dark photoperiod.

#### 2.1.1 Upper and lower critical thermal limits

Specimens (8 ≤ *N* ≤ 10) of each crab species were directly transferred from their acclimation temperatures to higher or lower temperatures in an incubator or thermal bath, for 6 h, to establish the species’ upper (UL_50_) and lower critical limits (LL_50_). Three or four different, consecutive temperatures above and below the respective control condition were chosen, such that mortality was greater than 50% in at least one temperature and less than 50% in another.This 6‐hr exposure period represents the mean emergence time of a crab at low tide and thus exposure to the greatest thermal amplitude occurring during the tidal cycle. Crabs were examined every 30 min and were considered “dead” if they could no longer right themselves after 30 sec. The critical thermal limits define the temperatures at which 50% of the crabs lose their key motor functions, being unable to right themselves when placed in a supine position, revealing disorganized locomotor activity. In this case, organismic functions are affected as “the animal loses its ability to escape from conditions that will promptly lead to its death” (Cowles & Bogert, 1944). Critical thermal limits were determined by probit analysis (Finney, 1971), adjusting the percentage survival in a linear regression model.

#### 2.1.2 Phylogenetic analysis

A partial fragment of the mitochondrial 16S ribosomal gene (≈590 base pairs) was used as a molecular marker. This gene has been widely used in studies of phylogenetic inferences in decapod crustaceans (*e.g*., Mantelatto, Pardo, Pileggi, & Felder, 2009; Mantelatto, Robles, Schubart, & Felder, 2009; Pileggi & Mantelatto, 2010; Schubart, Cuesta, Diesel, & Felder, 2000). Twelve new sequences were obtained, one for each species. All sequences obtained were submitted to NCBI GenBank (Table 1). Other species of Brachyura were included in the alignment to provide a more consistent analysis (Table 1) following previous phylogenetic methodologies (Tsang et al., 2014). Some species of Anomura were used as outgroups (Table 1).

**Table 1:** Species used in the molecular phylogenetic analysis with their respective localities, catalogue numbers, and GenBank access numbers

Species	Locality	Catalogue number	GenBank access number
Brachyura			
***Acanthocyclus albatrossis***	Ushuaia, Argentina	CCDB 5738	KT279704
***Aratus pisonii***	Ilha de Itamaracá, Brazil	CCDB 5732	KT279694
***Armases rubripes***	Rio Grande, Brazil	CCDB 5735	KT279701
***Cardisoma ganhumi***	Ipojuca, Brazil	CCDB 3927	KT279695
***Goniopsis cruentata***	Tamandaré, Brazil	MZUSP 29928	KT279696
***Halicarnicus planatus***	Ushuaia, Argentina	CCDB 5739	KT279705
***Neohelice granulata***	Rio Grande, Brazil	CCDB 5736	KT279702
***Ocypode quadrata***	Ilha de Itamaracá, Brazil	CCDB 5733	KT279697
***Pachygrapsus transversus***	Ilha de Itamaracá, Brazil	CCDB 5734	KT279698
***Uca maracoani***	Sirinhaem, Brazil	CCDB 2999	KT279699
***Uca uruguayensis***	Rio Grande, Brazil	CCDB 5737	KT279703
***Ucides cordatus***	Ipojuca, Brazil	CCDB 4467	KT279700
*Calappa gallus*	–	–	EU920916
*Charybdis hellerii*	Venezuela	ULLZ 4631	DQ407666
*Cymonomus diogenes*	–	CP3652	KJ132531
*Dorippe quadridens*	–	ZRC2008.0064	KJ132536
*Epialtus brasiliensis*	Brazil	CCDB 2373	KC695763
*Euphylax robustus*	Panama	ULLZ 8670	KT279706
*Lauridromia dehanii*	–	NTOU B00006	KJ132568
*Megaesthesius yokoyai*	–	–	KJ132589
*Menippe rumphii*	–	MSLKHC Mrum	KJ132579
*Micropanope scultipes*	Brazil	CCDB 5271	KT279707
*Mithraculus forceps*	–	–	KJ132583
*Parthenope longimanus*	–	–	KJ132604
*Persephona townsendi*	Panama	ULLZ 13931	JX102071
*Pilumnus longicornis*	–	MSLKHC BR140 Pllon	KJ132612
*Platypodiella spectabilis*	Belize	ULLZ 11077	KF682989
*Ranina ranina*	–	NTOU B00012	KJ132629
*Sesarma rectum*	Brazil	CCDB 3669	KT279708
*Trichodactylus dentatus*	–	SMF 32763	KJ132642
Anomura			
*Calcinus tibicen*	Brazil	–	DQ369940
*Coenobita violascens*	Taiwan	NTOU A00841	KJ132519
*Lithodes turritus*	Taiwan	NTOU A01107	KJ132573
*Lomis hirta*	Melbourne, Australia	–	AF436052
*Hippa adactyla*	Taiwan	NMNS 4368‐027	KJ132557
*Petrolisthes galathinus*	Colon, Panama	–	AF260638

Species whose thermal critical limits were characterized here are given in bold. CCDB (Coleção de Crustáceos do Departamento de Biologia, Faculdade de Filosofia, Ciências e Letras de Ribeirão Preto, Universidade de São Paulo), MSLKHC (Simon F. S. Li Marine Science Laboratory, School of Life Sciences, The Chinese University of Hong Kong), MZUSP (Museu de Zoologia of the Universidade de São Paulo), NMNS (National Museum of Natural Science, China), NTOU (National Taiwan Ocean University), SMF (Senckenberg Museum, Germany), ULLZ (University of Louisiana at Lafayette Zoological Collection), ZRC (Zoological Reference Collection of the National University of Singapore).

Genomic DNA was extracted using Chelex resin (Estoup, Largiader, Perrot, & Chourrout, 1996) or the salt extraction method (Mantelatto, Robles, Biagi, & Felder, 2006) followed by PCR amplification (Sambrook, Fritsch, & Maniatis, 1989). The following primers were employed: 16L9 (5′‐CGCCTGTTTATCAAAAACAT‐3′) and 16H9 (5′‐CCGGTCTGAACTCAGATCACGT‐3′) (Palumbi & Benzie, 1991), 16SL2 (5′‐TGCCTGTTTATCAAAAACAT‐3′) (Schubart et al., 2000) or 1471 (5′‐CCTGTTTANCAAAAACAT‐3′) (Shih, Naruse, & Ng, 2010) and 1472 (5′‐AGATAGAAACCAACCTGG‐3′) (Schubart et al., 2000). The thermal cycling protocol consisted of initial denaturing for 5 min at 95°C; annealing for 40 cycles—45 s at 95 °C, 45 s at 48 °C, 1 min at 72 °C; and a final extension for 3 min at 72 °C. PCR products were purified using the SureClean Plus kit and sequenced employing the ABI Big‐Dye Terminator Mix (Applied Biosystem, Carlsba, CA, USA) on an ABI 3730xl DNA Analyzer (Applied Biosystems automated sequencer) following the manufacturer's protocol.

A consensus sequence for two strands was edited and constructed using BIOEDIT 7.0.5 software (Hall, 1999). Sequences were aligned using ClustalW (Thompson, Higging, & Gibson, 1994) with an interface to BIOEDIT (Hall, 1999) employing default parameters.

Phylogenetic reconstructions were performed using the maximum likelihood search method (Felsenstein, 1973, 1981) in RAxML 7.2.7 (Randomized A(x)ccelerated Maximum Likelihood) (Stamatakis, 2006) implemented on the CIPRES (Cyberinfrastructure for Phylogenetic Research) system (http://www.phylo.org). The substitution model used was GTR + ∫ + I [General Time Reversible (Tavaré, 1986) + Gama + invariables sites] as specified by RAxML. Topology consistency was measured using a rapid bootstrap method (1,000 replicates) (Stamatakis, Hoover, & Rougemont, 2008) and only confidence values greater than 50% were reported. The topologies were visualized and edited using MEGA 5.1 software. The phylogeny obtained is provided in Figure 1 and was pruned to match the number of species (12) for which thermal tolerances were characterized. Topology and branch lengths among species were maintained, as is standard practice for comparative evaluations employing nonmissing trait data (Kembel et al., 2010).

#### 2.1.3 Comparative analyses

Comparisons among closely related species, when performed using an appropriate phylogeny, real measure of branch lengths and good fitting model of evolution, reduce type I error, decreasing the probability of detecting spurious correlations owing to uncontrolled factors. This also reduces type II error, adding stronger statistical power allowing the detection of correlated evolution between ecological/physiological and environmental traits (Diniz‐Filho, 2001; Garland & Adolph 1994; Garland et al., 2005; Rezende & Diniz‐Filho, 2012). Thus, the molecular phylogeny hypothesized here, with its real estimates of branch lengths under different models of evolution, confers a statistical power of testing to phylogenetically based analyses equivalent to that found when employing traditional statistical methods (Garland and Adolph 1994).

Phylogenetic patterns of thermal tolerance and microhabitat temperature were described employing an autocorrelation analysis, using Moran's *I* autocorrelation coefficient for three distance classes, and are presented as a phylogenetic correlogram (Diniz‐Filho, 2001; Gittleman & Kot, 1990). Each class shows the phylogenetic signal between pairs of species as a function of phylogenetic distance, from lower to higher hierarchical levels. Moran's *I* varies from −1.0 to +1.0: Significant positive values demonstrate high similarity between closely related species; significant negative values suggest that related species are dissimilar. The analysis was performed using the Phylogenetic Analysis in Macroecology application (PAM version 0.9 beta, Rangel et al., 2015).

Hypotheses regarding the effect of zoogeographical province and microhabitat temperature on the evolution of upper and lower critical thermal limits were tested using a phylogenetic generalized least squares (PGLS) model. Using categorical and quantitative predictors (PGLS ANCOVA), phylogenetically correlated residual variation among species (Garland & Ives, 2000; Grafen, 1989; Lavin, Karasov, Ives, Middleton, & Garland, 2008) is assumed, which is ideal for comparative studies concerning mean values for species. To avoid interference from uncontrolled environmental conditions (Wildt & Olli 1978), MHT was treated as a covariable, which removes its effects, corrects mean values, and improves the power of hypothesis testing regarding the effect of the discrete predictor (province). Simultaneously, we estimated selection strength (α) of the Ornstein–Uhlenbeck (O‐U) model of evolution, in which the minimum and maximum values of a trait are limited, stabilizing them toward a central point of variation (Butler & King, 2004; Diniz‐Filho, 2001; Nunn, 2011; Revell, 2010). When α is null, covariance among species is a consequence of stochastic evolutionary changes, or results from randomly shifting optima driven by natural selection, as modeled by Brownian motion (Butler & King, 2004; Hansen, Pienaar, & Orzack, 2008). The Holm–Bonferroni *post hoc* procedure was used to compare multiple means, P‐values being corrected by phylogenetic simulations (Revell, 2012). Analyses were performed using the R environment (R Core Team 2015), and its *nlme* (Pinheiro, Bates, DebRoy, Sarkar & R Core Team, 2016) and *ape* (Paradis, Claude, & Strimmer, 2004) packages, with the minimum significance level set at *p* = .05.

## 3 Results and Discussion

The microhabitat temperatures (MHT) of the crab species from the Brazilian and Argentinian zoogeographical provinces were similar, but higher than those of the Magellanic species. Both MHT and the upper critical thermal limits (UL_50_) showed strong phylogenetic signal in the first distance class, although there was no correlation at more inclusive hierarchical levels. The lower critical thermal limits (LL_50_) show no phylogenetic signal. Province seems to have affected the evolution of thermal tolerance, and only UL_50_ is dependent on MHT. LL_50_ differed markedly among all provinces while UL_50_ was similar between the two northernmost provinces compared to the Magellanic province.

**Figure 2:**
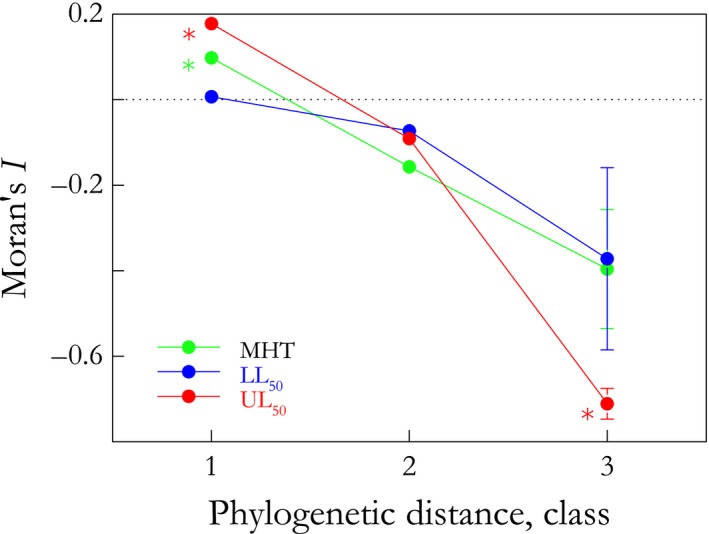
Phylogenetic correlogram over three distance classes, using Moran's *I* coefficients (± SEM), for microhabitat temperatures (MHT) and lower (LL_50_) and upper (UL_50_) critical thermal limits of 12 eubrachyuran crab species from three different zoogeographical provinces (see Figure 1). Horizontal line at *I* = 0.0 indicates no correlation with phylogeny. LL_50_ does not correlate with phylogeny at any hierarchical level, revealing labile evolution; MHT and UL_50_ exhibit strong phylogenetic signal at the species level, which decreases with phylogenetic distance, suggesting that closely related species share similar thermal niches and mechanisms of high‐temperature tolerance

Microhabitat temperatures are the consequence of complex interactions among the distribution of macrophytes, distance from the subtidal zone, and structural diversity of the habitat. Crab species from the Brazilian province were obtained under the shadow of mangrove trees, and on sandy beaches or rocky shores, which increases the interspecific variability of their MHT, from ≈22 °C for *Ucides cordatus* and ≈36 °C for *Uca maracoani* (Table 2, Figure 2). Crabs from the Argentinian province are distributed throughout salt marshes, with bushy vegetation in drier areas, dominated by the cord‐grass *Spartina* sp. in frequently flooded regions. Given the lesser structural diversity of salt marshes, mean MHT showed little variation: between ≈26 °C for *Neohelice granulata,* ≈27 °C for *Armases rubripes*, and ≈29 °C for *Uca uruguayensis* (Table 2, Figure 2). The two intertidal crabs from the Magellanic province, *Acanthocyclus albatrossis* and *Halicarcinus planatus*, were collected under rocks from beaches at low syzygy tide, at a temperature of ≈1.5 °C. An effect of zoogeographical province on MHT was detected (phylANOVA, *F* = 33.1, *p* ≤ .01): The Magellanic species (1.5 ± 0.1 °C) differed from those of the Argentinian (27.3 ± 0.6 °C) and Brazilian (27.2 ± 1.4 °C) provinces (phyHolm‐Bonferroni, *p* ≤ .01), with no difference between the thermal niches of the latter two provinces (phyHolm‐Bonferroni, *p* = .99).

**Table 2:** Habitat characteristics, microhabitat temperatures (7 ≤ *N* ≤15), and lower (LL_50_) and upper (UL_50_) critical thermal limits for 12 eubrachyuran crab species collected from three zoogeographical provinces along the eastern coast of South America

Species	Substrate	MHT	LL_50_	UL_50_
		(°C)		
Brazilian province				
*Aratus pisonii*	Mangrove trees	27.0 ± 0.6	12.8	36.9
*Cardisoma guanhumi*	Sandy clay, supralittoral	26.2 ± 0.4	13.4	38.6
*Goniopsis cruentata*	Mangrove mud, under roots	28.2 ± 0.8	12.8	36.0
*Ocypode quadrata*	Sandy beaches, supralittoral	21.8 ± 0.8	13.8	36.2
*Pachygrapsus transversus*	Rocky shores	29.1 ± 1.1	13.8	36.2
*Uca maracoani*	Mangrove mud, deep burrows	36.3 ± 0.5	12.8	38.6
*Ucides cordatus*	Mangrove mud, under roots	22.1 ± 1.5	15.5	39.0
Argentinian province				
*Armases rubripes*	Under rocks, among *Spartina*	27.0 ± 1.2	8.7	36.1
*Neohelice granulata*	Sandy clay, among *Spartina*	25.5 ± 0.7	6.5	36.7
*Uca uruguayensis*	Sandy beaches, mesolittoral	29.3 ± 1.4	10.4	39.3
Magellanic province				
*Acanthocyclus albatrossis*	Under rocks, mesolittoral	1.5 ± 0.1	−0.2	29.0
*Halicarcinus planatus*	Under rocks, mesolittoral	1.4 ± 0.1	−0.1	23.0

All temperature data are given in °C.

As regards phylogenetic pattern, MHT displayed strong phylogenetic signal at the species level, as revealed by the significant and positive Moran's *I* values for first distance class (*I* = 0.1 ± 0.0, *p* ≤ .05) (Figure 2). Thus, closely related species appear to share similar thermal niches. However, no correlation with phylogeny was detected as phylogenetic distance increases (I→0). This is reinforced by the lack of difference between the mean MHT values for the Brazilian and Argentinian provinces (both ≈27 °C). This suggests the existence of selective pressures (as described by the pGLS ANOVA) together with inertial components (as detected by the autocorrelation analysis) in the evolution of the thermal niche of the eubrachyuran crabs examined here.

Both upper and lower critical thermal limits appear to display different phylogenetic patterns (Figure 2). LL_50_ seemed to show neither similarities nor dissimilarities between pairs of closely or distantly related species, although there was a tendency toward negative autocorrelation. UL_50_ appeared to be strongly structured at the more apical and deeper hierarchical levels: Closely related species tended to share similar upper limits, while more distant species exhibited significant differences (Figure 2). This pattern of decreasing phylogenetic correlation with increasing phylogenetic distance typifies the Brownian motion model of evolution, as the evolutionary changes in UL_50_ are constant and associated with divergence time or phylogenetic distance among species.

**Figure 3:**
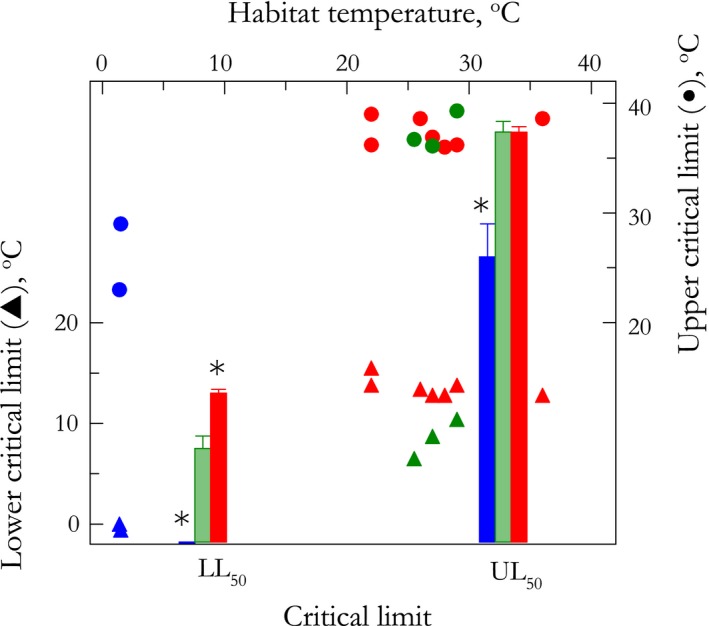
Lower (LL_50_) and upper (UL_50_) critical thermal limits for 12 eubrachyuran crab species from three zoogeographical provinces on the eastern coast of South America (see Figure 1). Limits were established after 6‐h direct exposure to the annual mean seawater temperatures of each province (sensu Boschi, 2000a; see Section 2 for details). Critical limits (bars) are given as the mean ± *SEM* (2 ≤*N* ≤ 7). LL_50_, 13.6 ± 0.4 °C for the Brazilian (*red*), 8.5 ± 1.1 °C for Argentinian (*green*), and −0.2 ± 0.1 °C for the Magellanic (*blue*) provinces; UL_50_, 37.4 ± 0.5°C, 37.4 ± 1.0 °C, and 26.0 ± 3.0 °C, respectively. *Significantly different from the Argentinian province (phyHolm‐Bonferroni, *p* ≤ .05). Filled triangles (LL_50_) and circles (UL_50_) represent the critical thermal limits for each species plotted against microhabitat temperature (upper *X*‐axis). The evolution of both limits is affected by zoogeographical province under O‐U process. Microhabitat temperature is not associated with LL_50_, but does correlate with UL_50_

The evolution of both critical thermal limits was affected by zoogeographical province (pGLS ANCOVA, 11.3 ≤ *F* ≤ 140.2, *p* ≤ .05) under O‐U process, in which random evolutionary changes are stabilized by a restriction force (α = 7.9 for LL_50_; α = 1.9 for UL_50_). The LL_50_ differed among the species from all provinces (phyHolm‐Bonferroni, *p* ≤ .01): 13.6 ± 0.4 °C for the Brazilian, 8.5 ± 1.1 °C for the Argentinian, and −0.2 ± 0.1 °C for the Magellanic provinces (Table 1, Figure 3). The UL_50_ was lower in the Magellanic province (26.0 ± 3.0 °C) than in the Argentinian (37.4 ± 1.0 °C) and Brazilian (37.4 ± 0.5 °C) provinces (phyHolm‐Bonferroni, *p* ≤ .01), the latter two being similar (phyHolm‐Bonferroni, *p* = .99). However, MHT has no effect on LL_50_ (pGLS ANCOVA, *F* = 1.6, *p* = .24), differently from the significant linear relationship seen for UL_50_ (pGLS ANCOVA, *F* ≥ 8.8; *p* ≤ .02). There was no significant interaction between MHT and province for either limit (pGLS ANCOVA, 3.1 ≤ *F*≤ 3.3; *p* = .11).

The evolution of LL_50_ is more plastic than that of UL_50_, as suggested by the lower phylogenetic signal and the effect of province under O‐U process showing stronger selection strength, both suggesting a role for natural selection in shaping the evolution of the lower thermal limit. The transformational changes produced randomly were counterbalanced by a significant restriction force, maintaining variation in LL_50_ values to one or more adaptive peaks, possibly close to the mean value of each zoogeographical province: ≈13.5°C, ≈8.5 °C, and ≈0.2 °C for the Brazilian, Argentinian, and Magellanic provinces, respectively. Further, under O‐U modeling, historical events tend to be relatively insignificant, corroborating the observed lack of phylogenetic pattern for the lower limit. Thus, there is an asymmetry in the evolution of both critical limits: LL_50_ is more sensitive to temperature than UL_50_, owing to the effects of thermal province under O‐U process, while UL_50_ shows a higher level of phylogenetic inertia owing to the strong autocorrelation pattern.

Fiddler crabs (*Uca*) from tropical regions exhibit higher upper thermal limits than representatives from subtropical and temperate zones (Vernberg & Tashian, 1959; Vernberg & Vernberg, 1967). Differences in UL_50_ in the crabs *Carcinus maenas* and *Cancer pagurus* reflect their vertical position within the intertidal zone: higher on the shore for *C. maenas* (UL_50_ = 31–35 °C) and a lower position for *C. pagurus* (UL_50_ = 21–31 °C) (Cuculescu et al., 1995). The most complete macroevolutionary scenario for upper thermal limits proposed to date refers to anomurans (*Petrolisthes*): In species occupying the upper intertidal, and in those from tropical zones, the evolution of UL_50_ is linked to surface water temperatures and maximal microhabitat temperatures (Stillman, 2002; Stillman & Somero, 2000). With regard to LL_50_, populations of fiddler crabs *U. pugilator*,* U. pugnax*, and *U. uruguayensis* from cooler microhabitats survive longer at lower temperatures than do populations from warmer regions, and than other congeneric species of exclusively tropical distribution (Demeusey, 1957; Tashian, 1956; Vernberg & Tashian, 1959; Vernberg & Vernberg, 1967).

The evolution of thermal tolerance in invertebrates depends on the proportion and types of fatty acids in their cell membranes (Hazel & Williams, 1990; Kates, Moldoveanu, & Stewart, 1993), and on a positive balance between the capability for oxygen supply and oxygen demand (Pörtner, 2001, 2002). A higher percentage of polyunsaturated fatty acids in winter than in summer appears to underpin the physical properties of the membranes affecting regulation of intracellular osmolality, as transport enzyme kinetics are dependent on membrane fluidity and on intracellular ionic composition (Hazel & Williams, 1990; Murata & Wada, 1995). This phenomenon is known as the “homeoviscous adaptation” (Hazel & Williams, 1990; Kates et al., 1993). Further, maintenance of aerobic scope is linked to an efficient cardiorespiratory system in supplying sufficient oxygen for mitochondrial demands, in addition to the heat‐shock response and antioxidant capability in providing protection against protein denaturation and reactive oxygen species (Peck, Webb, & Bailey, 2004; Pörtner, 2001; Zielinski & Pörtner, 1996). Thus, strong selection pressure at the cellular and subcellular levels may explain the lower LL_50_ observed in the crab species from Magellanic province, as well as the more elevated UL_50_ seen in the eubrachyurans from the two northernmost provinces. These findings reveal the homoplastic nature of the physiological mechanisms of thermal tolerance arrayed against acute thermal challenge.

Thus, the limits of thermal tolerance in eubrachyuran crabs appear to manifest distinct physiological constraints, are subject to different environmental pressures, and show unique evolutionary histories. The upper and lower critical thermal limits are informative descriptors of the discontinuous distribution of crabs throughout the intertidal zone of eastern South America, as they reflect different functional properties of an integrated system. The macroevolutionary landscape explored here suggests an asymmetrical scenario for eubrachyuran thermal tolerance, because the critical thermal limits are differentially inherited and environmentally driven.

## Conflict of Interest

None declared.

## References

[ece32741-bib-0001] Addo‐Bediako, A. , Chown, S. L. , & Gaston, K. J. (2000). Thermal tolerance, climatic variability and latitude. Proceedings of the Royal Society of London, Series B: Biological Sciences, 267, 739–745. 10819141 10.1098/rspb.2000.1065 PMC1690610

[ece32741-bib-0002] Boschi, E. E. (2000a). Species of decapod crustaceans and their distribution in the American marine zoogeographic Provinces. Revista de Investigación y Desarrollo Pesquero, 13, 7–136.

[ece32741-bib-0003] Boschi, E. E. (2000b). Biodiversity of marine decapod brachyurans of the Americas. Journal of Crustacean Biology, 20, 337–342.

[ece32741-bib-0004] Boschi, E. E. , & Gavio, M. A. (2005). On the distribution of decapod crustaceans from the Magellan Biogeographic Province and the Antartic region. Scientia Marina, 69, 195–200.

[ece32741-bib-0005] Briggs, J. C. (1974). Marine zoogeography. New York: McGraw‐Hill.

[ece32741-bib-0006] Butler, M. A. , & King, A. A. (2004). Phylogenetic comparative analysis: A modeling approach for adaptive evolution. The American Naturalist, 164, 683–695. 10.1086/426002 29641928

[ece32741-bib-0007] Calosi, P. , Bilton, D. T. , & Spicer, J. I. (2008). Thermal tolerance, acclimatory capacity and vulnerability to global climate change. Biology Letters, 4, 99–102. 17986429 10.1098/rsbl.2007.0408 PMC2412917

[ece32741-bib-0008] Calosi, P. , Bilton, D. T. , Spicer, J. I. , Votier, S. C. , & Atfield, A. (2010). What determines a species’ geographical range? Thermal biology and latitudinal range size relationships in European diving beetles (Coleoptera: Dytiscidae). Journal of Animal Ecology, 79, 194–204. 19761459 10.1111/j.1365-2656.2009.01611.x

[ece32741-bib-0009] Carcelles, A. R. , & Williamson, S. I. (1951). Catalogo de los moluscos de la provincia magallanica. Revista del Instituto Nacional de Investigación de las Ciencias Naturales, 2, 255–383.

[ece32741-bib-0010] Compton, T. J. , Rijkenberg, M. J. A. , Drent, J. , & Piersma, T. (2007). Thermal tolerance ranges and climate variability: A comparison between bivalves from differing climates. Journal of Experimental Marine Biology and Ecology, 352, 200–211.

[ece32741-bib-0011] Cooke, A. H. (1895). Mollusca In Harmer S. F. & Shipley A. E. (Eds.), The Cambridge natural history. London: MacMillan.

[ece32741-bib-0012] Cowles, R. B. , & Bogert, C. M. (1944). A preliminary study of the thermal requirements of desert reptiles. Bulletin of the American Museum of Natural History, 83, 265–296.

[ece32741-bib-0013] Cruz, F. B. , Fitzgerald, L. A. , Espinoza, R. E. , & Schulte, J. A. (2005). The importance of phylogenetic scale in tests of Bergmann's and Rapoport's rules: Lessons from a clade of South American lizards. Journal of Evolutionary Biology, 18, 1559–1574. 16313468 10.1111/j.1420-9101.2005.00936.x

[ece32741-bib-0014] Cuculescu, M. , Hyde, D. , & Bowler, K. (1995). Temperature acclimation of marine crabs: Changes in plasma membrane fluidity and lipid composition. Journal of Thermal Biology, 20, 207–222.

[ece32741-bib-0015] Demeusey, N. (1957). Respiratory metabolism of the fiddler crab *Uca pugilator* from two different latitudinal populations. Biological Bulletin, 113, 245–253.

[ece32741-bib-0016] Diniz‐Filho, J. A. F. (2001). Phylogenetic autocorrelation under distinct evolutionary processes. Evolution, 55, 1104–1109. 11475046 10.1111/j.0014-3820.2001.tb00630.x

[ece32741-bib-0017] Estoup, A. , Largiader, C. R. , Perrot, E. , & Chourrout, D. (1996). Rapid one‐tube extraction for a reliable PCR detection of fish polymorphic markers and transgenes. Molecular Marine Biology and Biotechnology, 5, 295–298.

[ece32741-bib-0018] Felsenstein, J. (1973). Maximum likelihood and minimum‐steps methods for estimating evolutionary trees from data on discrete characters. Systematic Biology, 22, 240–249.

[ece32741-bib-0019] Felsenstein, J. (1981). Evolutionary trees from DNA sequences: A maximum likelihood approach. Journal of Molecular Evolution, 17, 368–376. 7288891 10.1007/BF01734359

[ece32741-bib-0020] Felsenstein, J. (1985). Phylogenies and the comparative method. The American Naturalist, 125, 1–15. 10.1086/703055 31094602

[ece32741-bib-0021] Finney, D. J. (1971). Probit analysis, 3rd ed Cambridge: Cambridge University Press.

[ece32741-bib-0101] Garland, T. , & Adolph, S. C. (1994). Why not do two‐species comparative studies: limitations on inferring adaptation. Physiological and Biochemical Zoology, 67, 797–828.

[ece32741-bib-0022] Garland, T. Jr , Bennett, A. F. , & Rezende, E. L. (2005). Phylogenetic approaches in comparative physiology. Journal of Experimental Biology, 208, 3015–3035. 16081601 10.1242/jeb.01745

[ece32741-bib-0023] Garland, T. Jr , & Ives, I. R. (2000). Using the past to predict the present: Confidence intervals for regression equations in phylogenetic comparative methods. The American Naturalist, 155, 346–364. 10.1086/303327 10718731

[ece32741-bib-0024] Gittleman, J. L. , & Kot, M. (1990). Adaptation: Statistics and a null model for estimating phylogenetic effects. Systematic Zoology, 39, 227–241.

[ece32741-bib-0025] Grafen, A. (1989). The phylogenetic regression. Philosophical Transactions of the Royal Society of London. Series B, Biological Sciences, 326, 119–157. 2575770 10.1098/rstb.1989.0106

[ece32741-bib-0026] Hall, T. A. (1999). BioEdit: A user‐friendly biological sequence alignment editor and analysis program for Windows 95/98/NT. Nucleic Acids Symposium Series, 41, 95–98.

[ece32741-bib-0027] Hansen, T. F. , Pienaar, J. , & Orzack, S. H. (2008). A comparative method for studying adaptation to a randomly evolving environment. Evolution, 62, 1965–1977. 18452574 10.1111/j.1558-5646.2008.00412.x

[ece32741-bib-0028] Harvey, P. H. , & Pagel, M. D. (1991). The comparative method in evolutionary biology. Oxford: Oxford University Press.

[ece32741-bib-0029] Hazel, J. R. , & Williams, E. E. (1990). The role of alterations in membrane lipid‐composition in enabling physiological adaptation of organisms to their physical‐environment. Progress in Lipid Research, 29, 167–227. 2131463 10.1016/0163-7827(90)90002-3

[ece32741-bib-0030] Heise, K. , Puntarulo, S. , Nikinmaa, M. , Abele, D. , & Pörtner, H. O. (2006). Oxidative stress during stressful heat exposure and recovery in the North Sea eelpout (*Zoarces viviparus*). Journal of Experimental Biology, 209, 353–363. 16391357 10.1242/jeb.01977

[ece32741-bib-0031] Jensen, G. C. , & Armstrong, D. A. (1991). Intertidal zonation among congeners: Factors regulating distribution of porcelain crabs *Petrolisthes* spp. (Anomura: Porcellanidae). Marine Ecology Progress Series, 73, 47–60.

[ece32741-bib-0032] Kates, N. , Moldoveanu, M. , & Stewart, L. C. (1993). On the revised structure of the major phospholipid of *Halobacterium salinarium* . Biochimica et Biophysica Acta, 1169, 46–53. 8334149 10.1016/0005-2760(93)90080-s

[ece32741-bib-0033] Kellermann, V. , Loeschcke, V. , Hoffmann, A. A. , Kristensen, T. N. , Fløjgaard, C. , David, J. R. , ··· Overgaard, J. (2012). Phylogenetic constraints in key functional traits behind species'climate niches: Patterns of desiccation and cold resistance across 95 *Drosophila* species. Evolution, 66, 3377–3389. 23106704 10.1111/j.1558-5646.2012.01685.x

[ece32741-bib-0034] Kellermann, V. , Overgaard, J. , Hoffmann, A. A. , Fløjgaard, C. , Svenning, J. C. , & Loeschcke, V. (2012). Upper thermal limits of *Drosophila* are linked to species distributions and strongly constrained phylogenetically. Proceedings of the National Academy of Sciences of the United States of America, 109, 16228–16233. 22988106 10.1073/pnas.1207553109 PMC3479592

[ece32741-bib-0035] Kembel, S. W. , Cowan, P. D. , Helmus, M. R. , Cornwell, W. K. , Morlon, H. , Ackerly, D. D. , ··· Webb, C. O. (2010). Picante: R tools for integrating phylogenies and ecology. Bioinformatics, 26, 1463–1464. 20395285 10.1093/bioinformatics/btq166

[ece32741-bib-0036] Lavin, S. R. , Karasov, W. H. , Ives, A. R. , Middleton, K. M. , & Garland, T. Jr (2008). Morphometrics of the avian small intestine, compared with nonflying mammals: A phylogenetic perspective. Physiological and Biochemical Zoology, 81, 526–550. 18754728 10.1086/590395

[ece32741-bib-0037] Lutterschmidt, W. I. , & Hutchison, V. H. (1997). The critical thermal maximum: History and critique. Canadian Journal of Zoology, 75, 1561–1574.

[ece32741-bib-0038] Mantelatto, F. L. , Pardo, L. M. , Pileggi, L. G. , & Felder, D. L. (2009). Taxonomic re‐examination of the hermit crab species *Pagurus forceps* and *Pagurus comptus* (Decapoda: Paguridae) by molecular analysis. Zootaxa, 2133, 20–32.

[ece32741-bib-0039] Mantelatto, F. L. , Robles, R. , Biagi, R. , & Felder, D. L. (2006). Molecular analysis of the taxonomic and distributional status for the hermit crab genera *Loxopagurus* Forest, 1964 and *Isocheles* Stimpson, 1858 (Decapoda, Anomura, Diogenidae). Zoosystema, 28, 495–506.

[ece32741-bib-0040] Mantelatto, F. L. , Robles, R. , Schubart, C. D. , & Felder, D. L. (2009). Molecular phylogeny of the genus *Cronius* Stimpson, 1860, with reassignment of *C. tumidulus* and several American species of *Portunus* to the genus *Achelous* De Haan, 1833 (Brachyura: Portunidae) In Martin J. M., Crandall K. A. & Felder D. L. (Eds.), Crustacean issues: Decapod crustacean phylogenetics (pp. 537–551). Boca Raton: Taylor & Francis/CRC Press.

[ece32741-bib-0041] Manush, S. M. , Pal, A. K. , Chatterjee, N. , Das, T. , & Mukherjee, S. C. (2004). Thermal tolerance and oxygen consumption of *Macrobrachium rosenbergii* acclimated to three temperatures. Journal of Thermal Biology, 29, 15–19.

[ece32741-bib-0042] Murata, N. , & Wada, H. (1995). Acyl‐lipid desaturases and their importance in the tolerance and acclimatization to cold of cyanobacteria. The Biochemical Journal, 308, 1–8. 7755550 10.1042/bj3080001 PMC1136835

[ece32741-bib-0043] Nunn, C. L. (2011). The comparative approach in evolutionary anthropology and biology. Chicago: The University of Chicago Press.

[ece32741-bib-0044] Paine, R. T. (1974). Intertidal community structure: Experimental studies on the relationship between a dominant competitor and its principal predator. Oecologica, 15, 93–120. 10.1007/BF00345739 28308255

[ece32741-bib-0045] Palumbi, S. R. , & Benzie, J. (1991). Large Mitochondrial DNA differences between morphologically similar penaeid shrimp. Molecular Marine Biology and Biotechnology, 1, 27–34. 1669002

[ece32741-bib-0046] Paradis, E. , Claude, J. , & Strimmer, K. (2004). Ape: Analyses of phylogenetics and evolution in R language. Bioinformatics, 20, 289–290. 14734327 10.1093/bioinformatics/btg412

[ece32741-bib-0047] Peck, L. S. , Webb, K. E. , & Bailey, D. M. (2004). Extreme sensitivity of biological function to temperature in Antarctic marine species. Functional Ecology, 18, 625–630.

[ece32741-bib-0048] Pileggi, L. G. , & Mantelatto, F. L. (2010). Molecular phylogeny of the freshwater prawn genus *Macrobrachium* (Decapoda, Palaemonidae), with emphasis on the relationships among selected American species. Invertebrate Systematics, 24, 194–208.

[ece32741-bib-0049] Pinheiro, J. , Bates, D. , DebRoy, S. , Sarkar, D. & R Core Team (2016). nlme: Linear and Nonlinear Mixed Effects Models. R package version 3.1‐128.

[ece32741-bib-0050] Pörtner, H. O. (2001). Climate change and temperature dependent biogeography: Oxygen limitation of thermal tolerance in animals. Naturwissenschaften, 88, 137–146. 11480701 10.1007/s001140100216

[ece32741-bib-0051] Pörtner, H. O. (2002). Climate variations and the physiological basis of temperature dependent biogeography: Systemic to molecular hierarchy of thermal tolerance in animals. Comparative Biochemistry and Physiology – Part A, 132, 739–761. 10.1016/s1095-6433(02)00045-4 12095860

[ece32741-bib-0103] R Core Team (2015). R: A Language and Environment for Statistical Computing. Vienna, Austria: R Foundation for Statistical Computing Available online at http://www.R-project.org/.

[ece32741-bib-0052] Rangel, T. F. , Colwell, R. K. , Graves, G. R. , Fucikova, K. , Rahbek, C. , & Diniz‐Filho, J. A. (2015). Phylogenetic uncertainty revisited: Implications for ecological analyses. Evolution, 69, 1301–1312. 25800868 10.1111/evo.12644

[ece32741-bib-0053] Ravaux, J. , Léger, N. , & Rabet, N. (2012). Adaptation to thermally variable environments: Capacity for acclimation of thermal limit and heat shock response in the shrimp *Palaemonetes varians* . Journal of Comparative Physiology, 182, 899–907. 22547175 10.1007/s00360-012-0666-7

[ece32741-bib-0104] Revell, L. J. (2010). Phylogenetic signal and linear regression on species data. Methods in Ecology and Evolution, 1, 319–329.

[ece32741-bib-0054] Revell, L. J. (2012). Phytools: An R package for phylogenetic comparative biology (and other things). Methods in Ecology and Evolution, 3, 217–223.

[ece32741-bib-0055] Rezende, E. L. , & Diniz‐Filho, J. A. F. (2012). Phylogenetic analyses: Comparing species to infer adaptations and physiological mechanisms. Comprehensive Physiology, 2, 645–674. 10.1002/cphy.c100079 23728983

[ece32741-bib-0056] Sambrook, J. , Fritsch, E. F. , & Maniatis, T. (1989). In vitro amplification of DNA by the Polymerase Chain Reaction In Sambrook J., Fritsch E. F., & Maniatis T. (Eds.), Molecular cloning: A laboratory manual. New York: Cold Spring Harbor Laboratory Press.

[ece32741-bib-0057] Schubart, C. D. , Cuesta, J. A. , Diesel, R. , & Felder, D. L. (2000). Molecular phylogeny, taxonomy, and evolution of nonmarine lineages within the American grapsoid crabs (Crustacea: Brachyura). Molecular Phylogenetics and Evolution, 15, 179–190. 10837150 10.1006/mpev.1999.0754

[ece32741-bib-0058] Selvakumar, S. , & Geraldine, P. (2005). Heat shock protein induction in the freshwater prawn *Macrobrachium malcolmsonii*: Acclimation influenced variations in the induction temperatures for Hsp70. Comparative Biochemistry and Physiology – Part A, 140, 209–215. 10.1016/j.cbpb.2005.01.008 15748861

[ece32741-bib-0059] Shih, H. T. , Naruse, T. , & Ng, P. K. L. (2010). *Uca jocelynae* sp. nov., a new species of fiddler crab (Crustacea: Brachyura: Ocypodidae) from the Western Pacific. Zootaxa, 2337, 47–62.

[ece32741-bib-0060] Sommer, A. , Klein, B. , & Pörtner, H. O. (1997). Temperature induced anaerobiosis in two populations of the polychaete worm *Arenicola marina* (L). Journal of Comparative Physiology, 167, 25–35.

[ece32741-bib-0061] Stamatakis, A. (2006). RAxML‐VI‐HPC: Maximum likelihood‐based phylogenetic analyses with thousands of taxa and mixed models. Bioinformatics, 22, 2688–2690. 16928733 10.1093/bioinformatics/btl446

[ece32741-bib-0062] Stamatakis, A. , Hoover, P. , & Rougemont, J. (2008). A rapid bootstrap algorithm for the RAxML web servers. Systematic Biology, 57, 758–771. 18853362 10.1080/10635150802429642

[ece32741-bib-0063] Stillman, J. H. (2002). Causes and consequences of thermal tolerance limits in rocky intertidal porcelain crabs, genus *Petrolisthes* . Integrative and Comparative Biology, 42, 790–796. 21708777 10.1093/icb/42.4.790

[ece32741-bib-0064] Stillman, J. H. , & Somero, G. N. (2000). A comparative analysis of the upper thermal tolerance limits of eastern Pacific Porcelain crabs, genus *Petrolisthes*: Influences of latitude, vertical zonation, acclimation and phylogeny. Physiological and Biochemical Zoology, 73, 200–208. 10801398 10.1086/316738

[ece32741-bib-0065] Sunday, J. M. , Bates, A. E. , & Dulvy, N. K. (2010). Global analysis of thermal tolerance and latitude in ectotherms. Proceedings of the Royal Society of London B: Biological Sciences, 278, 1823–1830. 10.1098/rspb.2010.1295 PMC3097822 21106582

[ece32741-bib-0066] Tashian, R. E. (1956). Geographic variation in the respiratory metabolism and temperature coefficient in tropical and temperate forms of the fiddler crab, *Uca pugnax* . Zoologica, 41, 39–47.

[ece32741-bib-0067] Tavaré, S. (1986). Some probabilistic and statistical problems in the analysis of DNA sequences In Miura R. M. (Ed.), Lectures on Mathematics in the Life Sciences (pp. 57–86). Rhode Island: American Mathematical Society.

[ece32741-bib-0068] Thompson, J. D. , Higging, D. G. , & Gibson, T. J. (1994). CLUSTALW: Improving the sensitivity of progressive multiple sequence alignment through sequence weighting specific gap penalties and weight matrix choice. Nucleic Acids Research, 22, 4673–4680. 7984417 10.1093/nar/22.22.4673 PMC308517

[ece32741-bib-0069] Thurman, C. L. , Faria, S. C. , & McNamara, J. C. (2013). The distribution of fiddler crabs (*Uca*) along the coast of Brazil: Implications for biogeography of the western Atlantic ocean. Marine Biodiversity Records, 6, 1–21.

[ece32741-bib-0070] Tomanek, L. (2005). Two‐dimensional gel analysis of the heat‐shock response in marine snails (genus *Tegula*): Interspecific variation in protein expression and acclimation ability. Journal of Experimental Biology, 208, 3133–3143. 16081611 10.1242/jeb.01748

[ece32741-bib-0071] Tsang, L. M. , Schubart, C. D. , Ahyong, S. T. , Lai, J. C. Y. , Au, E. Y. C. , Chan, T. Y. , … Chu, K. H. (2014). Evolutionary history of true crabs (Crustacea: Decapoda: Brachyura) and the origin of freshwater crabs. Molecular Biology and Evolution, 31, 1173–1187. 24520090 10.1093/molbev/msu068

[ece32741-bib-0072] Vernberg, F. J. , & Tashian, R. E. (1959). Studies on the physiological variation between tropical and temperature zone fiddler crabs of the genus *Uca*. II. Oxygen consumption of whole organisms. Biological Bulletin, 117, 163–184.

[ece32741-bib-0073] Vernberg, F. J. , & Vernberg, W. B. (1967). Thermal lethal limits of southern hemisphere *Uca* crabs: Studies on the physiological variation between tropical and temperate zone fiddler crabs of the genus *Uca*. IX. Oikos, 18, 118–123.

[ece32741-bib-0105] Wildt, A. R. , & Ahtola, O. (1978). Analysis of covariance, Vol. 12 Beverly Hills: Sage.

[ece32741-bib-0074] Zielinski, S. , & Pörtner, H. O. (1996). Energy metabolism and ATP free‐energy change of the intertidal worm, *Sipunculus nudus*, below a critical temperature. Journal of Comparative Physiology, 166, 492–500.

